# Establishment of patient-derived organoids and a characterization-based drug discovery platform for treatment of pancreatic cancer

**DOI:** 10.1186/s12885-022-09619-9

**Published:** 2022-05-03

**Authors:** Sadanori Watanabe, Akitada Yogo, Tsuguteru Otsubo, Hiroki Umehara, Jun Oishi, Toru Kodo, Toshihiko Masui, Shigeo Takaishi, Hiroshi Seno, Shinji Uemoto, Etsuro Hatano

**Affiliations:** 1 DSK Project, Medical Innovation Center, Graduate School of Medicine, Kyoto University, Kyoto, Japan; 2 Cancer Research Unit, Sumitomo Pharma Co., Ltd, Osaka, Japan; 3 Department of Surgery, Graduate School of Medicine, Kyoto University, Kyoto, Japan; 4 Department of Gastroenterology and Hepatology, Graduate School of Medicine, Kyoto University, Kyoto, Japan

**Keywords:** Pancreatic cancer, Organoid, Peritoneal dissemination, Xenograft model, Compound screening

## Abstract

**Background:**

Pancreatic cancer is one of the most lethal tumors. The aim of this study is to provide an effective therapeutic discovery platform for pancreatic cancer by establishing and characterizing patient-derived organoids (PDOs).

**Methods:**

PDOs were established from pancreatic tumor surgical specimens, and the mutations were examined using a panel sequence. Expression of markers was assessed by PCR, immunoblotting, and immunohistochemistry; tumorigenicity was examined using immunodeficient mice, and drug responses were examined in vitro and in vivo.

**Results:**

PDOs were established from eight primary and metastatic tumors, and the characteristic mutations and expression of cancer stem cell markers and CA19–9 were confirmed. Tumorigenicity of the PDOs was confirmed in subcutaneous transplantation and in the peritoneal cavity in the case of PDOs derived from disseminated nodules. Gemcitabine-sensitive/resistant PDOs showed consistent responses in vivo. High throughput screening in PDOs identified a compound effective for inhibiting tumor growth of a gemcitabine-resistant PDO xenograft model.

**Conclusions:**

This PDO-based platform captures important aspects of treatment-resistant pancreatic cancer and its metastatic features, suggesting that this study may serve as a tool for the discovery of personalized therapies.

**Supplementary Information:**

The online version contains supplementary material available at 10.1186/s12885-022-09619-9.

## Background

Pancreatic cancer is a devastating disease and has an extremely poor prognosis, with a five-year overall survival rate of around 10% [1]. Despite current interventions such as gemcitabine/nab-paclitaxel or FOLFIRINOX (5-fluorouracil, leucovorin, irinotecan, and oxaliplatin), the response rates remain poor and relapse is frequently observed [2–4]. In addition, pancreatic cancer progresses without subjective symptoms and frequently leads to metastasis, which is not curable with any current therapies [5]. Thus, tools and models to identify more effective therapeutic regimens for individual patients are urgently needed.

During the last decade, the technology has been established to grow tissues in vitro in three dimensions, resembling organs. These so-called organoids can be grown from adult and embryonic stem cells and are able to self-organize into 3D structures that reflect the tissue of origin [6]. Since organoids can be established and expanded from primary patient materials, patient-derived organoids (PDOs) have been used as alternative resources to conventional cell lines in research for cancer therapies based on their advantage of preserving the characteristics of original patients [6]. In fact, studies on hepatobiliary and pancreatic organoids including pancreatic cancer have progressed rapidly [7–9]. Since PDOs are relatively easy to maintain compared to patient-derived xenograft models, multiple approaches including personalized medicine through profiling PDOs’ responsiveness to therapeutic agents and establishment of pathological models have been applied in the cancer field [10–13]. However, few studies have examined the therapeutic effects in in vivo xenotransplantation models, which is the preclinical stage of testing.

In the present study, we established pancreatic cancer organoids from patients including those from metastatic tumors, and identified the characteristics of these PDOs in vitro. We also established new in vivo evaluation models capturing the characteristics of the original malignant tumors in patients with these PDO lines. Finally, we conducted high-throughput compound screening using the PDOs and identified a compound effective for inhibiting tumor growth in vivo. These results confirmed the usefulness of PDO-based models for pancreatic cancer therapy.

## Material and methods

### Human pancreatic cancer samples

Surgically resected specimens were obtained from pancreatic cancer patients at Kyoto University Hospital. Analyses for human subjects were approved by the Ethical Committee of Kyoto University Hospital. All experiments have been conducted in accordance with the Declaration of Helsinki as well as the guidelines and regulations of the Committee.

### Organoid culture

Mouse pancreatic organoids (StemCell Technologies #70933) were cultured in PancreaCult Organoid Growth Medium (StemCell Technologies #06040) according to the manufacturer’s protocol.

Patient-derived pancreatic cancer organoids were established from fresh surgical specimens obtained from patients who underwent surgical resection at Kyoto University Hospital, approved by the Ethics Committees (R1281) and by the Ethical Committee of Sumitomo Pharma (2017–04). The pathological characteristics of the primary tumor are presented in Table 1. Primary tumor tissue samples were processed as previously reported, with some modifications [7, 8, 14]. Briefly, the cell aggregates were embedded in Matrigel (Corning, Cambridge, MA, USA) and covered by a medium composed of 50% L-WRN conditioned medium (ATCC) containing L-Wnt3A, R-spondin 3, and Noggin, consisting of Advanced DMEM/F12 (Invitrogen, Carlsbad, CA, USA), 5% FBS, 2 mmol/l L-Alanyl-L-Glutamine (Wako, Tokyo, Japan), 100 units/ml penicillin, 0.1 mg/ml streptomycin (Nacalai Tesque), 2.5 μg/ml Plasmocin prophylactic (Invitrogen), 10 μM Y-27632 (Tocris Bioscience), 1x B27 Supplement (Thermo Fisher Scientific, Waltham, MA, USA), 1 μM SB431542 (Tocris Bioscience), 100 ng/ml recombinant human fibroblast growth factor-basic (bFGF; Thermo Fisher Scientific), and 20 ng/ml recombinant human epidermal growth factor (EGF; Thermo Fisher Scientific). After confirming several passages of the PDOs, the organoids were also cultured with the following “complete medium” consisting of Advanced DMEM/F12 (Invitrogen, Carlsbad, CA, USA), 2 mM Glutamax-I (Wako, Tokyo, Japan), 10 mM HEPES (Thermo Fisher Scientific), 100 units/ml penicillin, 0.1 mg/ml streptomycin (Nacalai Tesque), 10 μM Y-27632 (Tocris Bioscience), 1x B27 Supplement (Thermo Fisher Scientific, Waltham, MA, USA), 1 μM inhibitor of transforming growth factor-β (TGF-β) type I receptor, SB431542 (Tocris Bioscience), 50 ng/ml Wnt3A(R&D systems), 500 ng/ml R-spondin-1 (Peprotech Inc), 100 ng/ml Noggin (R&D systems), 100 ng/ml bFGF (Peprotech Inc), and 50 ng/ml EGF (Peprotech Inc). For culture of *SMAD4*-mutants, Sph18–06 was cultured in the complete medium without SB431542 (Tocris Bioscience). The passage number of PDOs was as follows: for in vitro experiments, Sph18–02 (≥P19), Sph18–06 (≥P8), Sph18–14 (≥P23), Sph18–21 (≥P12), Sph18–25 (≥P12), Sph19–07 (≥P12), Sph19–14 (≥P10), Sph19–22 (≥P6); and for in vivo transplantation experiments, Sph18–02 (≥P25), Sph18–06 (≥P16), Sph18–14 (≥P31), Sph18–21 (≥P31), Sph18–25 (≥P28), Sph19–07 (≥P19), Sph19–14 (≥P15), Sph19–22 (≥P16). Cell proliferation of PDOs was examined by seeding the same number of cells in triplicate and counting the cell number at day 7 using a Countess II FL automated cell counter (Thermo Fisher Scientific). Bright field images of PDOs were taken on an inverted microscope system (Olympus, IX73, 10x or 20x objective lenses).

**Table 1:**
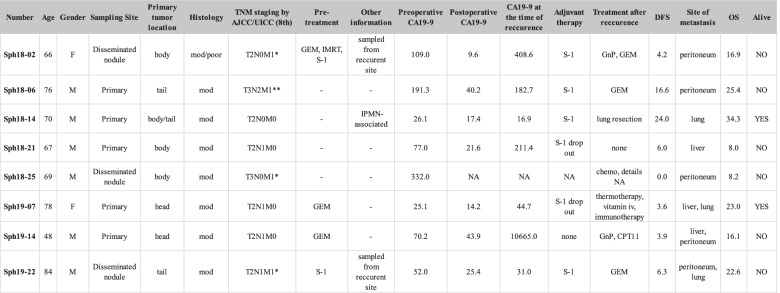
Additional data that provide clinical information about the established PDOs

Values in CA19–9 indicate U/mL. Values in DFS and OS indicate months

*Abbreviations: M* male, *F* female, *OS* overall survival, *DFS* disease-free survival, *mod* moderately differentiated adenocarcinoma, *poor* poorly differentiated adenocarcinoma, *AJCC* American joint committee on Cancer, *UICC* International Union against Cancer, *CA19–9* carbohydrate antigen 19–9, *GEM* gemcitabine, *IMRT* intensity-modulated radiotherapy, *S-1* Tegafur, Gimeracil, Oteracil potassium, *IPMN* Intraductal papillary mucinous neoplasm, *GnP* gemcitabine and nab-paclitaxel, *NA* data not available, *chemo* chemotherapy, *iv* intravenous injection, *CPT11* irinotecan

*M1 by peritoneal dissemination, **M1 by metastasis to para-aortic lymph node

For evaluation of effects of kinase inhibitor compounds on PDOs, cells of PDOs, Sph18–06 and Sph18–14, were dissociated, and the same number of cells (1 × 10^3^ cells/well) were plated in each of 384-well plates. After three days in culture, compounds from kinase inhibitor libraries (Selleck chemicals, L1200 and L2000) were added and further cultured for five days. Cell viability was examined by CellTiter-Glo 3D Reagent (Promega) according to the manufacturer’s instructions.

### Genetic mutation analysis of organoid lines

Organoids were dissociated, and DNA was isolated using the QIAamp DNA Mini Kit (Qiagen). Genetic mutations of PDOs were determined by next generation sequencing analysis using the Ion AmpliSeq 50-gene Cancer Hotspot Panel v2 with additional genes (Thermo Fisher Scientific, sequencing, mapping alignment, and annotation was outsourced to Takara Bio, Kusatsu, Japan). The panel included mutation hotspots for the following cancer-related genes: ABL1, AKT1, ALK, APC, ATM, BRAF, CDH1, CDKN2A, CSF1R, CTNNB1, EGFR, ERBB2, ERBB4, EZH2, FBXW7, FGFR1, FGFR2, FGFR3, FLT3, GNA11, GNAS, GNAQ, HNF1A, HRAS, JAK2, JAK3, IDH1, IDH2, KDR/VEGFR2, KIT, KRAS, MET, MLH1, MPL, NOTCH1, NPM1, NRAS, PDGFRA, PIK3CA, PTEN, PTPN11, RB1, RET, SMAD4, SMARCB1, SMO, SRC, STK11, TP53, VH, ARID1A, ARID2, ATRX, BAP1, DAXX, MEN1, RNF43, and TGFBR2. To preserve the quality of mutation detection, mutation candidates with homopolymer regions with lengths of ≥5 base pairs and those with sequencing coverage of 250 or fewer base pairs were excluded from analysis.

### Cell culture

The human pancreatic cancer cell lines, Panc-1 and BxPC-3 (ATCC), were cultured in DMEM or RPMI1640 supplemented with 10% FBS, 100 units/ml penicillin, and 0.1 mg/ml streptomycin (Nacalai Tesque) in a 5% CO_2_ incubator at 37 °C.

### Histochemical analysis

For immunohistochemical analysis, 3D-organoids were embedded in iPGell (Geno Staff) and fixed overnight in 4% paraformaldehyde (Nacalai Tesque). Tumor specimens were isolated and fixed overnight in 4% paraformaldehyde (Nacalai Tesque), embedded in paraffin and sectioned at a thickness of 3 or 4 μm. Sections were then deparaffinized, rehydrated, and stained with hematoxylin and eosin (HE). For immunohistochemical analyses, standard IHC procedures were performed in a BOND-RX automated immunostaining machine (Leica) according to the manufacturer’s instructions using anti-CD44 (1:600, Cell Signaling Technologies) and anti-CD133 (1:200, Abnova) antibodies. Images of the stained slides were captured and analyzed using an Aperio ImageScope (Leica, 20x objective lens) or inverted microscope systems (Olympus IX83 or Keyence BZ9000, 10x or 20x objective lenses) with the built-in software and ImageJ.

### Western blot and ELISA analysis

Samples were extracted using ice-cold RIPA buffer (Pierce) and separated using SDS-PAGE in 10–20% acrylamide gel (Wako). Proteins were transferred onto PVDF membranes using the iBlot dry transfer system (Invitrogen), and blocked using 3% skim milk (Wako). Proteins were incubated with the primary antibodies overnight at 4 °C. The primary antibodies used in this study were as follows: anti-PROM1/CD133 (1:1000, Abnova), anti-SOX2 (1:1000, Cell Signaling Technologies), anti-CD24 (1:500, Sigma Aldrich), anti-CA19–9 (1:500, Gene Tex). Samples were then incubated with horseradish peroxidase (HRP)-conjugated anti-mouse or anti-rabbit secondary antibodies (Jackson ImmunoResearch Labs, West Grove, PA, USA) for 60 minutes at room temperature. HRP-conjugated anti-beta actin (1:2000, Cell Signaling Technologies) antibody was also used as a loading control. Immunoreactive protein bands were identified with chemiluminescent HRP substrate (SuperSignal West Pico Plus Luminol/Enhancer Solution). Chemiluminescence signals were captured and analyzed using an ImageQuant LAS 500 (Cytiva) and ImageJ. For measurement of CA19–9 in cultured medium, same number of PDO cells (1 × 10^5^ cells / well) were embedded in Matrigel and cultured with 0.5 mL of the complete medium for 3 days, and the supernatant was collected and stored at − 80 °C until assay. The samples were analyzed using CA19–9 ELISA kit (RayBiotech) according to the manufacturer’s protocol.

### PCR array analysis

Total RNA was purified and DNase-treated using the RNeasy Mini Kit (Qiagen). PCR array analysis was performed using RT2 Profiler PCR array (Human Cancer Stem Cells) (PAHS-176Z) (SABiosciences, Frederick, MD, USA) according to the manufacturer’s protocol. Synthesis of cDNA was performed using iScript Reverse Transcription Supermix (Biorad, #1708840). Real time PCR was conducted using CFX-384 (Biorad). Fold changes relative to the control sample were calculated on the Qiagen Data Analysis Webportal (https://dataanalysis.qiagen.com/pcr/arrayanalysis.php). All signals were normalized to the levels of GAPDH and ACTB probes. RT^2^ Profile PCR Array Human Cancer Stem Cells (PAHS-176Z) was purchased from Qiagen. The assays were performed according to the manufacturer’s instructions.

### Flow cytometry

PDO samples were washed once with PBS (Nacalai Tesque), and then cells were dissociated with TrypLE Express (Thermo Fisher Scientific) and centrifuged. Single cell suspensions were washed once with Advanced DMEM/F12 (Thermo Fisher Scientific) containing 10% FBS. Cell pellets were resuspended in PBS containing 1% FBS and incubated for 30 min on ice with 10-fold dilution of the following antibodies: PE/Cy7 anti-CD44 (Bio-legend) and PE/Cy7 control IgG2b antibody (Bio-legend). Samples were passed through a 40 μm cell strainer (BD Biosciences) and resuspended in 500 μL incubation 1x PBS + 2% FBS to reach a final concentration of 10^6^ cells per 100 μl. Flow cytometry was carried out using a MACSQuant Analyzer 10 Flow Cytometer (Miltenyi Biotec). Cell debris was excluded by forward scatter pulse width and side scatter pulse width. Dead cells were excluded by labeling with LIVE/DEAD Fixable Near-IR Dead Cell Stain Kit (Thermo Fisher Scientific). The data were analyzed using software FlowJo (Tree Star, Ashland, OR, USA).

### Xenograft assay

All procedures for animal experiments were conducted in compliance with the ARRIVE guidelines and in accordance with the guidelines of the Animal Care and Use Committee at Sumitomo Pharma, Japan. Balb/c (Nude) mice were purchased from Charles River Laboratories Japan (Yokohama, Japan), and NOD/Shi-scid, IL-2RγKO Jic (NOG) mice were purchased from In-Vivo Science Inc. (Kawasaki, Japan). Mice were maintained in cages under standard conditions of ventilation, temperatures (20–26 °C), and lightning (Light/dark: 12 h / 12 h) and kept under observation for 1 week prior to experimentation. Drinking water and standard pellet diets were provided throughout the study. For subcutaneous grafts, 1 × 10^6^ or 3 × 10^5^ cell suspensions were resuspended in 50% Matrigel / 50% Hank’s balanced salt solution (HBSS) (Nacalai Tesque), and transplanted into the flanks of 6- to 8-week-old nude or NOG mice. Tumor size was measured with calipers once or twice a week after the injection. Volumes were calculated by applying the formula v = 0.5 × L × w × h, where v is volume, L is length, w is width and h is height. For the peritoneal dissemination model, PDOs were injected intraperitoneally with 1 or 3 × 10^6^ cells in 100 μL HBSS. For evaluation of the in vivo efficiency of gemcitabine and CHK1 inhibitor, prexasertib, mice with established subcutaneous tumors were randomized by splitting size-matched tumors into two groups (vehicle / gemcitabine or prexasertib), and the mice were subcutaneously administered 10 mg/kg prexasertib twice per day, three times a week. Gemcitabine was administered intraperitoneally at a dose of 30 or 60 mg/kg, two times a week.

### Statistics

All values are presented as mean ± SD unless otherwise stated. Statistical analysis was conducted using Prism v6 (GraphPad). Significant differences between groups were determined using a Student’s *t*-test. *P*-values < 0.05 were considered significant. Data distribution was assumed to be normal, but this was not formally tested.

## Results

### Establishment of organoids derived from pancreatic cancer tissue specimens and their characterization in vitro

**Fig. 1:**
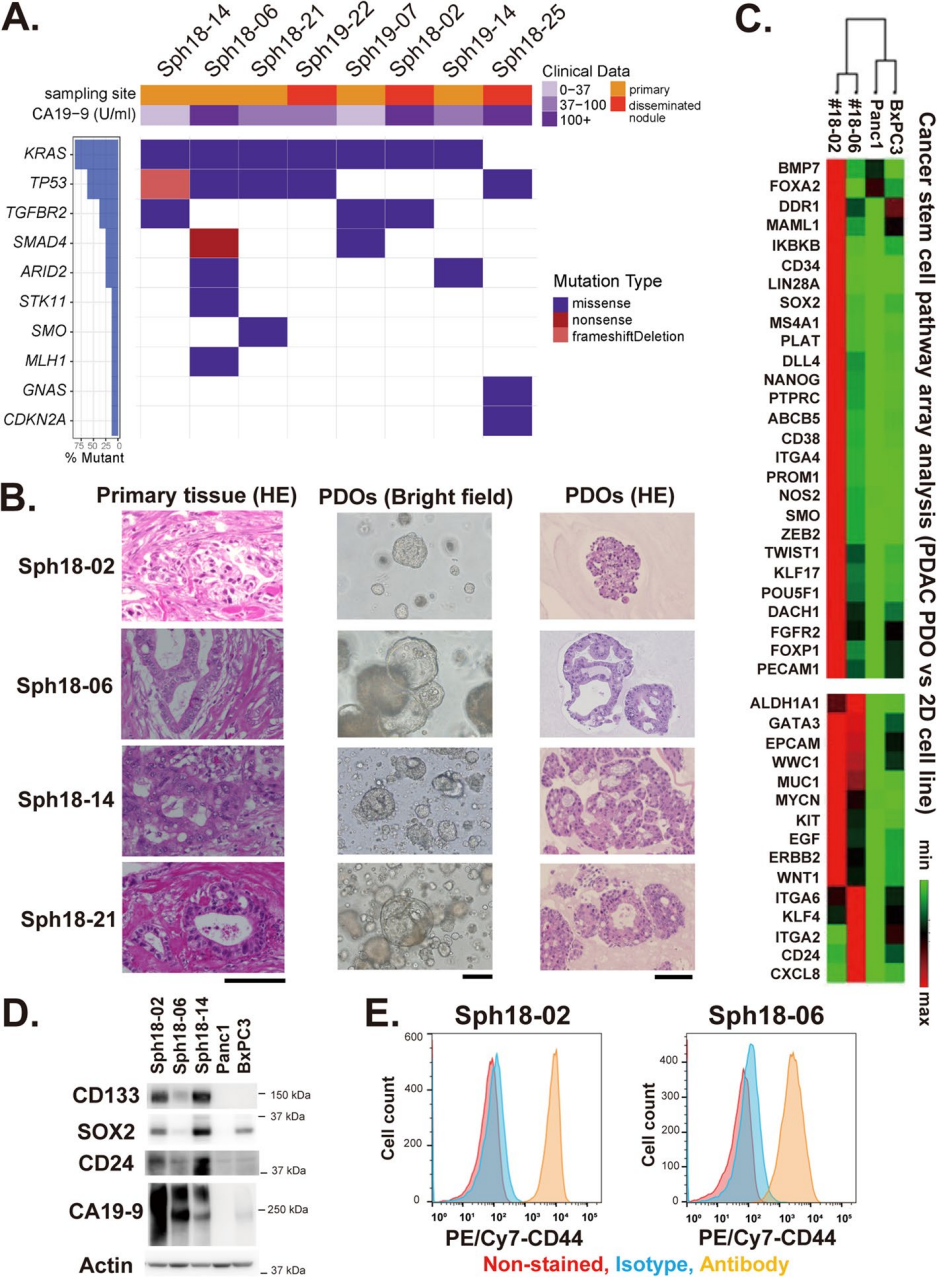
Establishment and characterization of pancreatic tumor organoids derived from primary and metastatic PDAC tissue specimens. **A**. Information about sampling sites and confirmed mutations in PDAC PDO lines. Shown are mutations confirmed by the ClinVar and COSMIC databases. See also Table S1. **B**. Histological characterization of pancreatic cancer PDOs. Shown are selected examples of specimens of primary tissues (left: HE-stained) and established organoids (middle: bright field, right: HE-stained). Scale bar, 100 μm. **C**. PCR microarray analysis of the expression of cancer stem cell genes in pancreatic cancer cell lines and PDOs. **D**. Western blotting analysis of CD133/PROM1, SOX2, CD24, CA19–9 in pancreatic cancer cell lines and PDOs. **E**. Flow cytometry analysis of PDOs. Histogram: CD44 (X-axis), cell count (Y-axis)

We established PDOs using surgically resected specimens of human pancreatic ductal adenocarcinoma (PDAC) based on the conditions of previous reports [7, 8, 14]. The overall success rate for establishing PDAC PDOs was 42% (8/19). These established PDO lines included those derived from the primary tumors as well as from peritoneal metastases (Fig. 1a and Table 1). To characterize the key genetic mutations, we sequenced the genomic regions of all eight PDOs covering the mutational hot spots of 50 cancer-related genes. Results showed typical mutations in the KRAS, TP53, SMAD4, and CDKN2A genes, all of which are common in pancreatic cancer (Fig. 1a and Table S1) [15]. These data indicated that the established PDOs were derived from pancreatic cancer epithelial cells, and neither mesenchymal nor endothelial cells.

Histologic examination of the PDAC PDOs confirmed characteristic features of cancer such as abnormal nuclear morphology and disruption of the striated linear ductal structure, which were not observed in normal pancreatic organoids (Fig. 1b) [7, 8]. Tubular ductal structures were observed in samples of PDOs, such as Sph18–06, 14, 21, and 19–22, which were similar to their original primary tumor tissues (Fig. 1b and Supplementary Fig. 1a).

It has been suggested that organoid culture could retain cellular hierarchies including tumor-initiating cancer stem cells (CSCs), in contrast to conventional culture methods [12, 16, 17]. As one of characterizations of PDOs, we analyzed the expression of CSC-related genes in two of our PDOs as well as two pancreatic cancer cell lines using an RT PCR array. This array consisted of 84 human cancer stem cell-related genes and multiple genes were highly expressed in the PDOs, especially in Sph18–02, compared with the cell lines (Fig. 1c). The expression of CD133/PROM1, one of the CSC markers reported in pancreatic cancer [18], was confirmed and particularly well expressed in PDOs (Fig. 1c, d, and Supplementary Fig. 1b). SOX2 was also highly expressed in Sph18–02, but the expression level in Sph18–06 was similar to the level in BxPC3 (Fig. 1c, d) [19]. Expression of other CSC markers including CD24 and CD44 was also observed in PDOs (Fig. 1d, e, and Supplementary Fig. 1b) [20].

We also examined a well-known prognostic biomarker of PDAC, carbohydrate antigen 19–9 (CA19–9) [21, 22], both in patients’ serum samples and PDOs, and confirmed that the expression is preserved in PDOs (Fig. 1a, d, and Supplementary Fig. 1c). These results together suggest that the established PDAC PDOs have characteristics of clinical pancreatic cancer in terms of mutation, histology, and expression of CSC-related markers.

### Creation of disease models of malignant pancreatic cancer using PDOs derived from primary and disseminated patient tumor samples

**Fig. 2:**
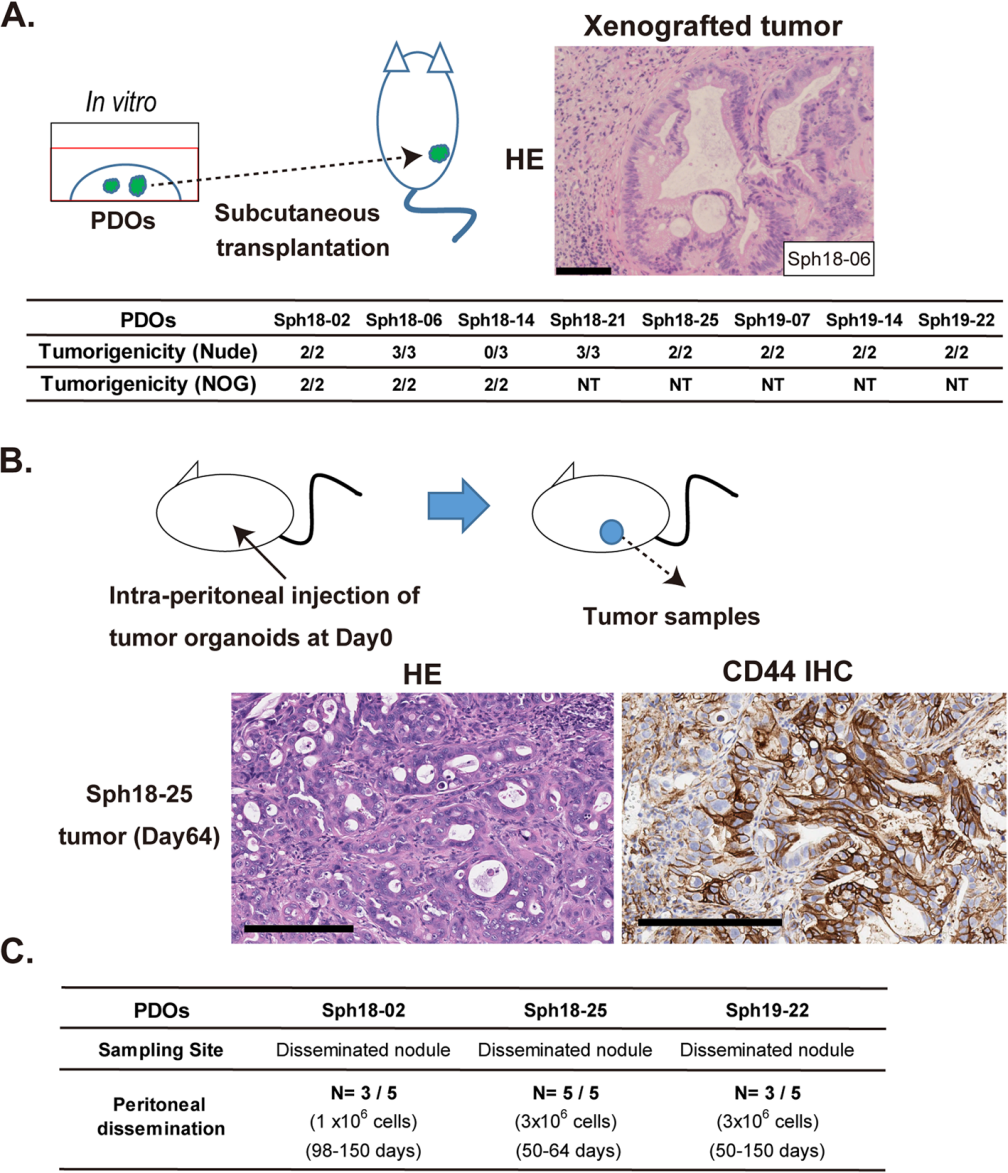
Creation of disease models of pancreatic cancer using PDOs derived from primary and disseminated patient tumor samples. **A**. Subcutaneous transplantation of PDOs and histological analysis of the formed tumors. Schematic representation of transplantation experiments (left top). Shown are selected examples of tumors of PDOs (right top: HE-stained). Scale bar, 100 μm. Subcutaneous tumorigenicity test of PDOs (bottom). Number of mice with tumors per total number of PDO-transplanted mice at 11 weeks after transplantation. NT, not tested. **B**. Schematic illustration of intra-peritoneal injection of PDOs into nude mice (top). Histochemical analysis of formed disseminated tumor nodule (left: HE-stained, right: CD44 IHC). Scale bar, 200 μm. **C**. Intraperitoneal tumor nodule formation in mice transplanted with PDOs. Three different PDOs were injected into nude mice (*N* = 5), and tumor nodule formation was evaluated

To examine the tumorigenic potential of PDAC PDOs, PDOs were subcutaneously transplanted into two types of immunodeficient mice (Fig. 2a). Although some PDOs did not form tumors in nude mice, tumor formation was observed in all cases with NOG mice (Fig. 2a and Supplementary Fig. 2a, b). The tumor growth speed was slower with Sph18–14 compared to Sph18–02, which is consistent with slow progression of the original patient’s IPMN-associated tumor (Supplementary Fig. 2b and Table 1). The histological images of the formed tumors reflected characteristics of pancreatic cancer, which is rich in stroma (Fig. 2a and Supplementary Figs. 1a, 2a), and also contained CD44-positive cells as observed in vitro (Supplementary Fig. 2c). These results suggest that these PDOs retain their tumorigenic potential and that these PDO xenograft (PDOX) models retain the clinically important characteristics of pancreatic cancer.

Since some of the PDAC PDOs were derived from tumors with peritoneal dissemination, we attempted to use these PDOs to establish a model of peritoneal dissemination by transplanting them into the peritoneum of nude mice (Fig. 2b). One of the PDOs, Sph18–25, was transplanted into the peritoneal cavity, and observation of the mice after 10 weeks revealed tumor mass formation in the peritoneal cavity (*N* = 5/5) (Fig. 2c). Other PDOs were also examined in a similar manner, and analysis further confirmed tumor formation within 50 to 100 days in mice transplanted with PDOs derived from disseminated nodules (Fig. 2c). In addition, presence of ascites was observed in a small number of cases related to Sph18–02 (*N* = 2/10, 1 or 3 × 10^6^ cells). In particular, tumor mass formation in the Sph18–25-transplanted mice was observed within a relatively early period and the mice died within 10 weeks (*N* = 3/5). These results suggest that PDOs derived from peritoneal disseminated nodules maintain their ability to proliferate and form peritoneal tumors, and also suggest that these PDOs are effectively recapitulating the characteristics of metastatic pancreatic cancer.

### Responses of PDOs to chemotherapy in vitro and in vivo

**Fig. 3:**
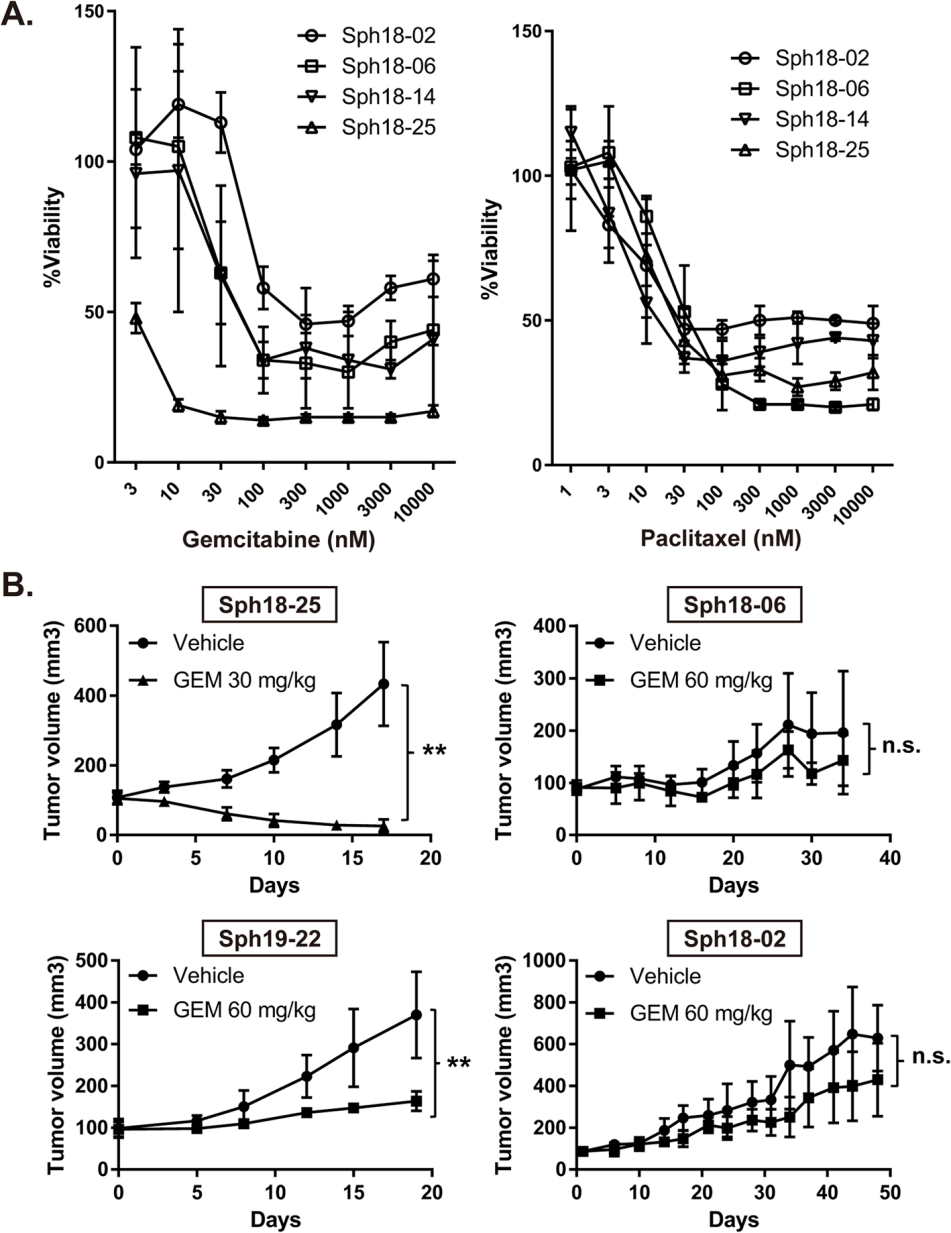
Response of PDOs and the xenograft model to chemotherapy. **A**. Dose-response curves after four days of treatment of PDOs with gemcitabine and paclitaxel. All of the experiments were carried out in triplicate, and data are represented as means ± SDs. **B**. Response of PDO xenograft model to gemcitabine. Four different PDOs, Sph18–02, 06, 25 and 19–22, were grafted subcutaneously in nude mice. Mice were treated with gemcitabine or vehicle twice a week at the indicated concentrations (*n* = 5). Results are shown as tumor volume (mm^3^ mean ± SD). **, *P* < 0.01; ns, not significant (two-tailed unpaired Student’s *t* test)

To examine responses of established PDOs to pancreatic cancer therapy, we treated them with commonly used therapeutic agents, such as gemcitabine and paclitaxel in vitro. Among the examined PDOs, Sph18–02 showed highest resistance to gemcitabine (Fig. 3a and Supplementary Fig. 3a). This result is consistent with the fact that Sph18–02 was derived from a tumor that relapsed after gemcitabine treatment (Table 1). Sph18–14 was less resistant to gemcitabine but showed relatively high resistance to paclitaxel, which may be related to its slow growth in vitro (Fig. 3a and Supplementary Fig. 3b).

Furthermore, the responses of PDO-derived tumors to gemcitabine were also examined using subcutaneous transplantation in vivo (Fig. 3b). Some of the PDO-derived tumors showed resistance to gemcitabine as seen in Sph18–02, while other tumors were sensitive as seen in Sph18–25. These results indicated that the response of PDOs to gemcitabine in vivo was as a whole correlated with the response in vitro*,* and further suggest that these PDOs can be used as a model reflecting the clinical phenotypes of pancreatic cancer.

### PDO-based drug screening using a kinase inhibitor library

**Fig. 4:**
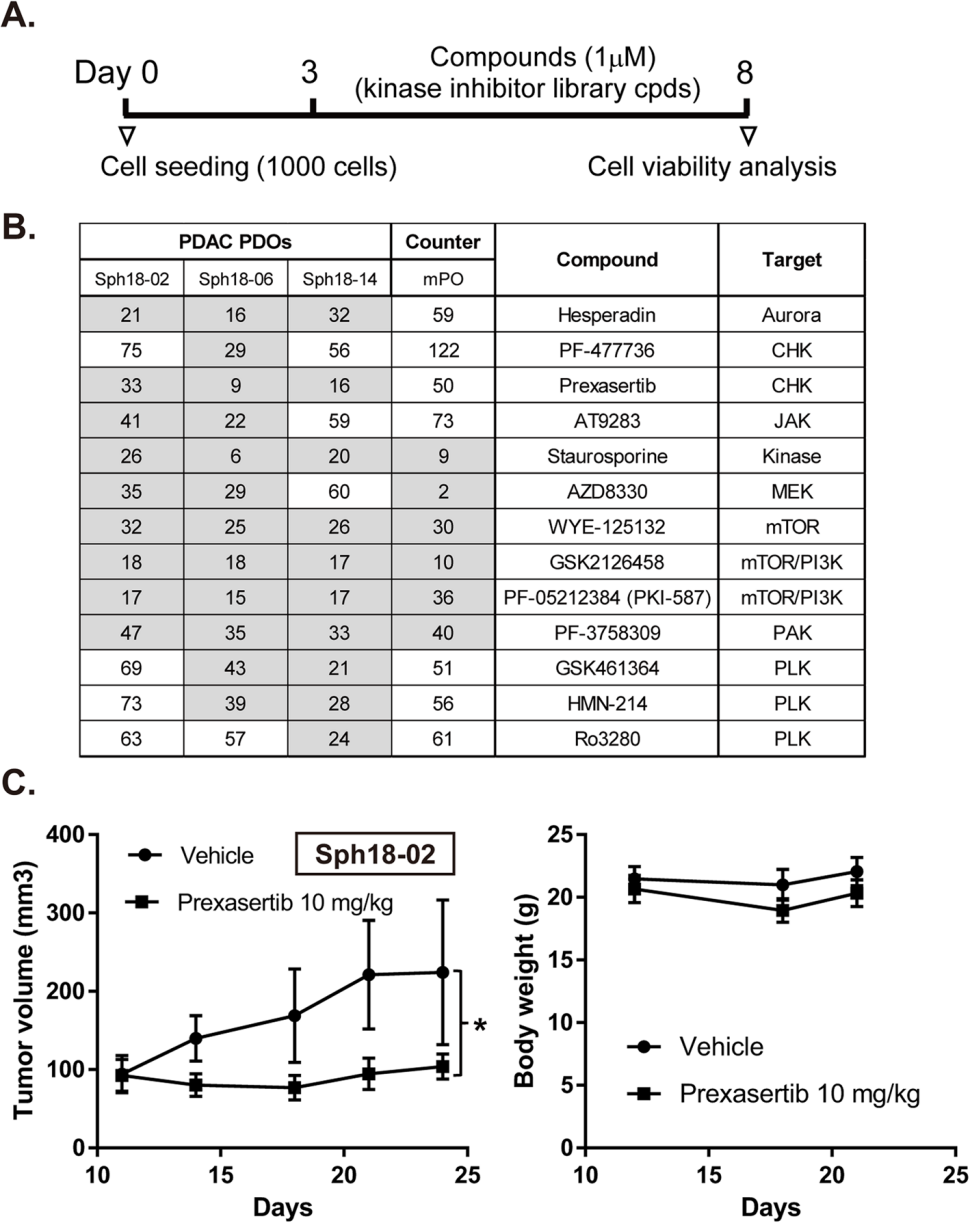
PDO-based drug screening using a kinase inhibitor library. **A**. Scheme of experiments for the treatment of PDOs with compounds. **B**. Summary of the responses of PDOs to different compounds used in the screening. Shown are values of percentage of viability versus DMSO. mPO, murine pancreatic organoid. Values below 50 are highlighted in gray. **C**. In vivo activity of the CHK1 inhibitor (prexasertib) in PDO Sph18–02 grafted subcutaneously in nude mice. Mice were treated with 10 mg/kg prexasertib or vehicle three times per week for two weeks (subcutaneous injection, *n* = 6). Results are shown as tumor volume (mm^3^ mean ± s.d.) (Left). *, *P* < 0.05 (two-tailed unpaired Student’s *t* test). Body weight change of mice bearing Sph18–02 PDO xenografts after treatment with 10 mg/kg prexasertib or vehicle (Right)

To further elucidate the usefulness of PDAC PDOs in drug discovery research, the response of PDOs to an inhibitor library was examined. PDOs were seeded in a 384-well format and treated with kinase inhibitor focused compounds (375 cpds) (Fig. 4a and Table S2), and viability was evaluated five days after treatment. The analysis revealed that several compounds reproducibly decreased the viability of the PDOs (Fig. 4b). Inhibitors of Aurora, CHK, mTOR, and PLK were found to be candidates for inhibiting the growth of these PDOs. Furthermore, Sph18–02, a relatively chemoresistant PDO line, was also examined with the use of the candidate compounds, and mTOR inhibitors effectively decreased its viability, but PLK1 had lesser effects on Sph18–02 than on the other two PDOs (Fig. 4b).

In order to confirm which of these compounds is less toxic to normal cells, the response of normal murine pancreatic organoids was examined (Fig. 4b). We found that some compounds, such as inhibitors of Aurora, CHK and PLK, had lower toxicity than the other compounds. When we focused on the compounds that were also effective against Sph18–02, inhibitors of Aurora and CHK were selected and had relatively lower toxic effects on normal organoids. To further confirm effectiveness, one of these compounds, prexasertib (a CHK1 inhibitor), was tested for its anti-tumor effects in a subcutaneous transplantation model using Sph18–02, which was resistant to gemcitabine treatment in the xenograft model. Treatment with a dose of 10 mg/kg effectively inhibited tumor growth, and severe body weight loss was not observed after treatment with this compound (Fig. 4c). These results together suggest that the established PDOs are useful for obtaining compounds with anti-tumor effects in pancreatic cancer therapy.

## Discussion

PDOs have been used more frequently in preclinical studies of cancer therapy since they retain a cellular hierarchy and are more reflective of clinical phenotypes [6, 12]. To maximize the usefulness of PDOs for personalized medicine or drug discovery research, it is crucial to understand the characteristics of PDOs in various aspects. Therefore, the comprehensive characterization of the PDOs in this study, including their characteristic mutations, tumorigenicity, and in vitro and in vivo drug responses, will enable us to efficiently utilize these PDOs in drug discovery research for pancreatic cancer, which will greatly advance the PDO-based drug discovery platform. This study has several important features.

First, established PDOs in this study reflected the histological and expressional features of PDAC in vitro and in vivo. Histological analysis of PDOs and the tumors revealed the presence of highly undifferentiated, well-differentiated duct-like tumor cells, and an enriched subcutaneous tumor stroma (Fig. 1b, Fig. 2a, b, Supplementary Figs. 1a and 2a). CA19–9 is a clinical biomarker for PDAC patients and has recently been shown to be an important factor that promotes rapid and aggressive pancreatic tumorigenesis [21, 22]. The expression of CA19–9 was confirmed in the PDOs, as reported previously in pancreatic cancer organoids [7], and this result is also in accordance with its detection in serum of the original patients, further supporting preservation of the clinical characteristics of PDAC (Fig. 1a, d, and Table 1). Expression analysis of PDOs also revealed the presence of CSC marker-positive cells (Fig. 1c, d, e), and a mixture of CD44-high and CD44-low cells was observed on immunohistochemical analysis of the PDO-derived tumors, further highlighting intra-tumoral heterogeneity (Fig. 2b and Supplementary Figs. 2c) [23]. In this study, the overall success rate was 42%, with limited efficiency and coverage of cancer subtypes. This rate is lower than the rates of previous reports, and might be due to conditions at the time of sampling from tissues [10, 11]. Indeed, the interval between tumor sampling and tissue extraction varied from 30 min to 2 h owing to surgical difficulty. Another important point is the composition of the culture medium. These problems could be solved by assessing conditions of clinical samples and optimizing the medium composition depending on the subtypes [9]. In order to understand the diversity of clinical cases more accurately and use PDOs more effectively, important future challenges include improving the efficiency with which PDOs are established, and increasing the number of PDOs.

Second, the established PDOs retained tumorigenic potential in two different transplantation models. The established PDAC PDOs include three dissemination-derived PDOs (Table 1), and this study is the first where a PDAC peritoneal dissemination model was established using PDOs (Fig. 2b, c). We confirmed that the PDOs established from the disseminated nodules had high tumorigenicity in the peritoneum; Sph18–25 proliferated particularly quickly in the peritoneum and had high lethality (Fig. 2c and data not shown). These data are consistent with the severe disease progression seen in the original patient (Table 1) and suggest that this PDO recapitulated the clinical phenotype of a malignant pancreatic cancer with peritoneal dissemination.

Third, the chemoresistance of the PDOs was defined in vitro and in vivo. Sph18–02 indeed had greater gemcitabine resistance than any other PDOs including Sph19–22, which was derived from a similar recurrent tumor, treated with S-1 (Fig. 3a, b, Table 1, S3). This suggests that each patient’s treatment history may have influenced chemotherapy resistance in the established PDOs. However, since this study has limitations in terms of number and coverage of individual cases, we could not exclude possible effects of combination treatment. In the original patient who provided Sph18–02, the disease progressed early on, even with gemcitabine treatment after recurrence (Table 1), suggesting a maintained phenotype in vitro and in vivo in PDO and highlighting the usefulness of this PDO line as a treatment-resistant model. Sph18–14 showed high CSC marker expression but without resistance to gemcitabine (Fig. 1d and Fig. 3a), suggesting that the high expression of CSC markers does not necessarily correlate with drug resistance.

Finally, a PDO-based screening platform to search for compounds with anti-tumor effects was established in this study (Fig. 4). Compared to previous studies, we applied compounds focusing on kinase inhibitors, and found potential candidate compounds that can be validated in vivo [10]. We found that several compounds were effective for PDAC PDOs, and among these, CHK1 inhibitors were included (Fig. 4b). A previous study showed that mutations in TP53 and vulnerability to DNA damage was associated with efficacy of a CHK1 inhibitor [24]. In fact, mutations in TP53 were detected in Sph18–06 and Sph18–14 (Fig. 1a). The other PDO Sph18–02, a recurrent line established after treatment with radiotherapy and gemcitabine, was also effectively suppressed by prexasertib treatment both in vitro and in vivo (Fig. 4b, c). One explanation for this efficacy may relate to higher dependence of Sph18–02 on CHK1-related intra-S and G2–M checkpoint DNA repair for cellular survival [25, 26]. Taken together, utilization of our characterized PDOs could thus comprehensively reveal that a CHK1 inhibitor is effective even for pancreatic cancer with gemcitabine-resistance. In addition, there are several reports showing that the combination of gemcitabine and prexasertib is effective for pancreatic cancer, which partly supports the validity of the results [26–29]. From a clinical perspective, trials of CHK1 inhibitors (Phase I or II) are ongoing in solid tumors, breast cancer and ovarian cancer, suggesting that CHK1 inhibition could also be effective in pancreatic cancer with selection of appropriate patients [30].

In conclusion, our established PDOs captured the key aspects of pancreatic cancer such as treatment resistance and peritoneal metastasis, and our characterization-based platform is expected to be useful for pancreatic cancer drug discovery research.

## Supplementary Information

**Additional file 1.**

**Additional file 2.**

**Additional file 3.**

**Additional file 4.**

## Data Availability

The datasets used and/or analyzed during the current study are available from the corresponding author on reasonable request.

## Abbreviations

PDOs: Patient-derived organoids
PCR: Polymerase chain reaction
PDAC: Pancreatic ductal adenocarcinoma
PDOX: PDO xenograft
CA19–9: Carbohydrate antigen 19–9
FOLFIRINOX: 5-fluorouracil, leucovorin, irinotecan, and oxaliplatin
FBS: Fetal bovine serum
FGF: Fibroblast growth factor
EGF: Epidermal growth factor
HE: Hematoxylin and eosin
RIPA: Radioimmunoprecipitation assay buffer
PVDF: Polyvinylidene Fluoride
HRP: Horse radish peroxidase
PBS: Phosphate buffered saline
HBSS: Hank’s balanced salt solution
NOG: NOD/Shi-scid, IL-2RγKO Jic SD Standard deviation
CSCs: Cancer stem cells
OS: Overall survival
DFS: Disease-free survival
AJCC: American Joint Committee on Cancer
UICC: International Union Against Cancer
GEM: Gemcitabine
IMRT: Intensity-modulated radiotherapy
S-1: Tegafur, Gimeracil, Oteracil Potassium IPMN Intraductal papillary mucinous neoplasm
GnP: Gemcitabine and nab-Paclitaxel
