# Supplementary material for: Establishment of patient-derived organoids and a characterization-based drug discovery platform for treatment of pancreatic cancer
Source: BMC Cancer. 2022 May 3;22:489. doi: 10.1186/s12885-022-09619-9 (PMC9063137; doi:10.1186/s12885-022-09619-9)
Supplement: Supplementary file 4 — Additional file 4. [file 12885_2022_9619_MOESM4_ESM.docx]

**SUPPORTING INFORMATION**

**Table S1**

Hotspot mutations in 58 oncogenes and tumor suppressor genes of PDO lines. The genomic regions covered by a customized hotspot gene panel (see Supporting information extended methods) were sequenced.

**Table S2**

Compounds used in this study

**Table S3**

Additional data for clinical pretreatment of the established PDOs

**Supplementary Figure 1,2,3,4**


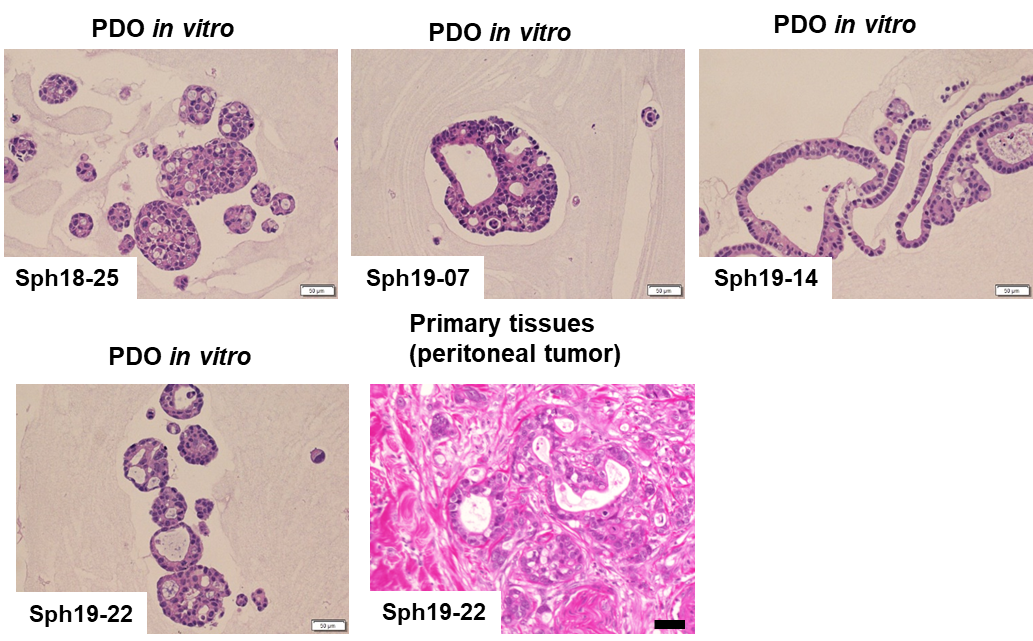
**Supplementary Figure 1.**

1. Shown are selected examples of specimens of established PDOs and primary tissues (HE-stained). Scale bars, 50 μm.


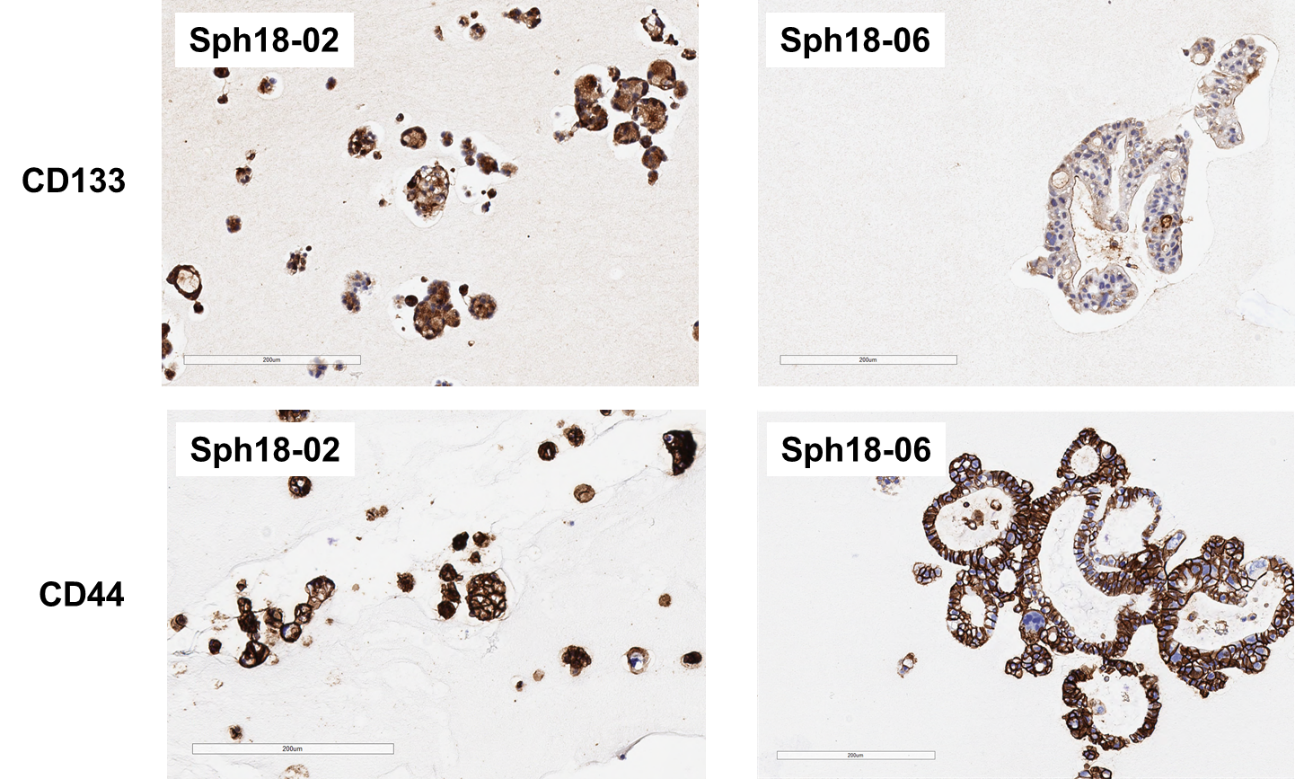


1. Immunohistochemical CD133 and CD44 staining of PDOs in vitro. Scale bar, 200 μm.


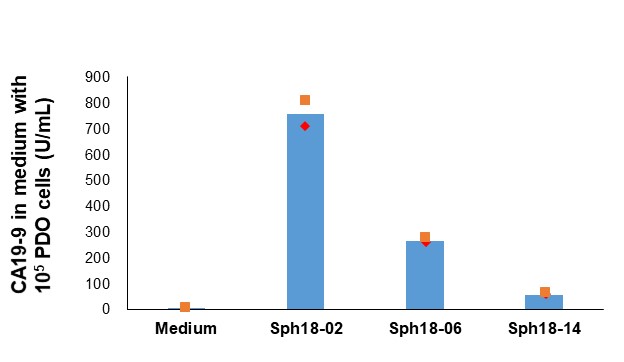


C. Measurement of CA19-9 levels in medium cultured with PDOs. PDOs were cultured for 3 days, and the levels of CA19-9 in the medium were measured by ELISA assay. CA19-9 levels (unit/mL, N=2, average) were adjusted with the number of cultured PDO cells.

**Supplementary Figure 2.**

**
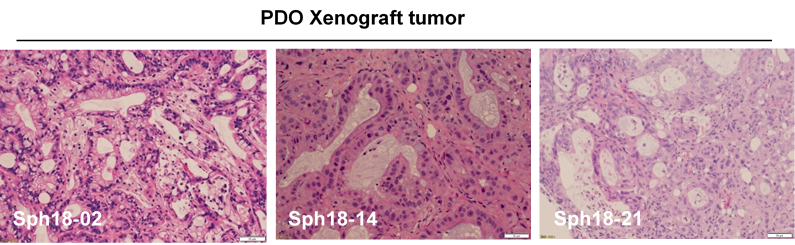
**

1. Histological characterization of PDO tumors grafted subcutaneously in nude mice (HE-stained). Scale bar, 50 μm.


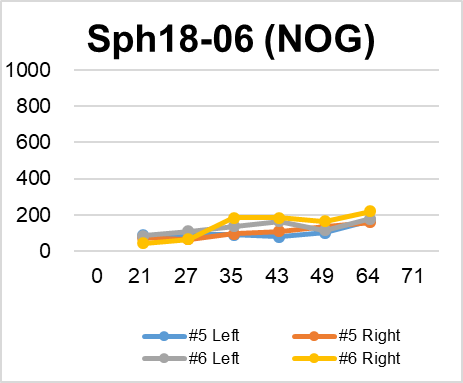

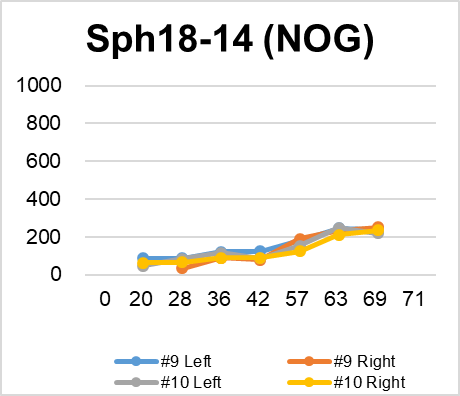

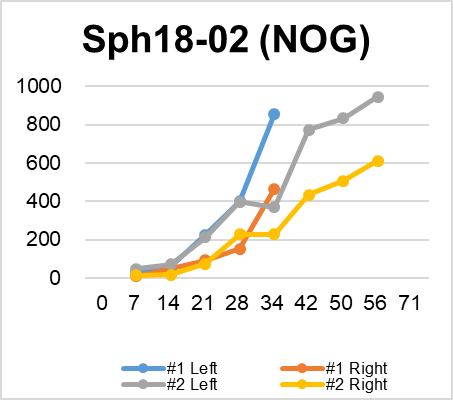


Days after transplantation

Days after transplantation

Days after transplantation

Subcutaneous transplantation

(Left: 1 x 10^6^ cells)

(Right: 3 x 10^5^ cells)

1. Growth of PDO tumors grafted subcutaneously in nude mice. Values shown are tumor volumes (mm^3^).


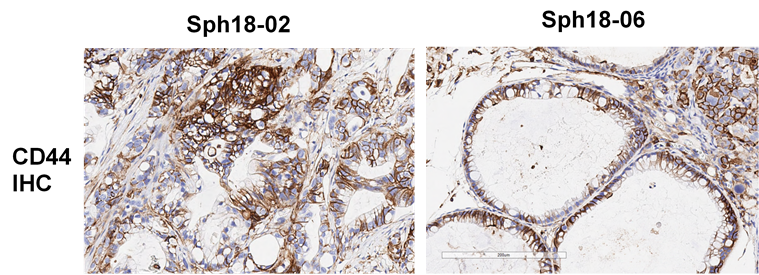


1. Immunohistochemical CD44 staining of PDO tumors grafted subcutaneously in nude mice. Scale bar, 200 μm.

**Supplementary Figure 3.**

**Gemcitabine (nM)**

**Paclitaxel (nM)**

1. Dose-response curves after four days of treatment of PDOs with gemcitabine and paclitaxel. All of the experiments were carried out in triplicate, and data are represented as means ± SDs.
2. Analysis of cell proliferation of PDOs. Cell numbers were counted after seven days of culture. All of the experiments were carried out in triplicate, and data are represented as means ± SDs.

**Supplementary Figure 4. Uncropped blot images of Fig.1d**

**
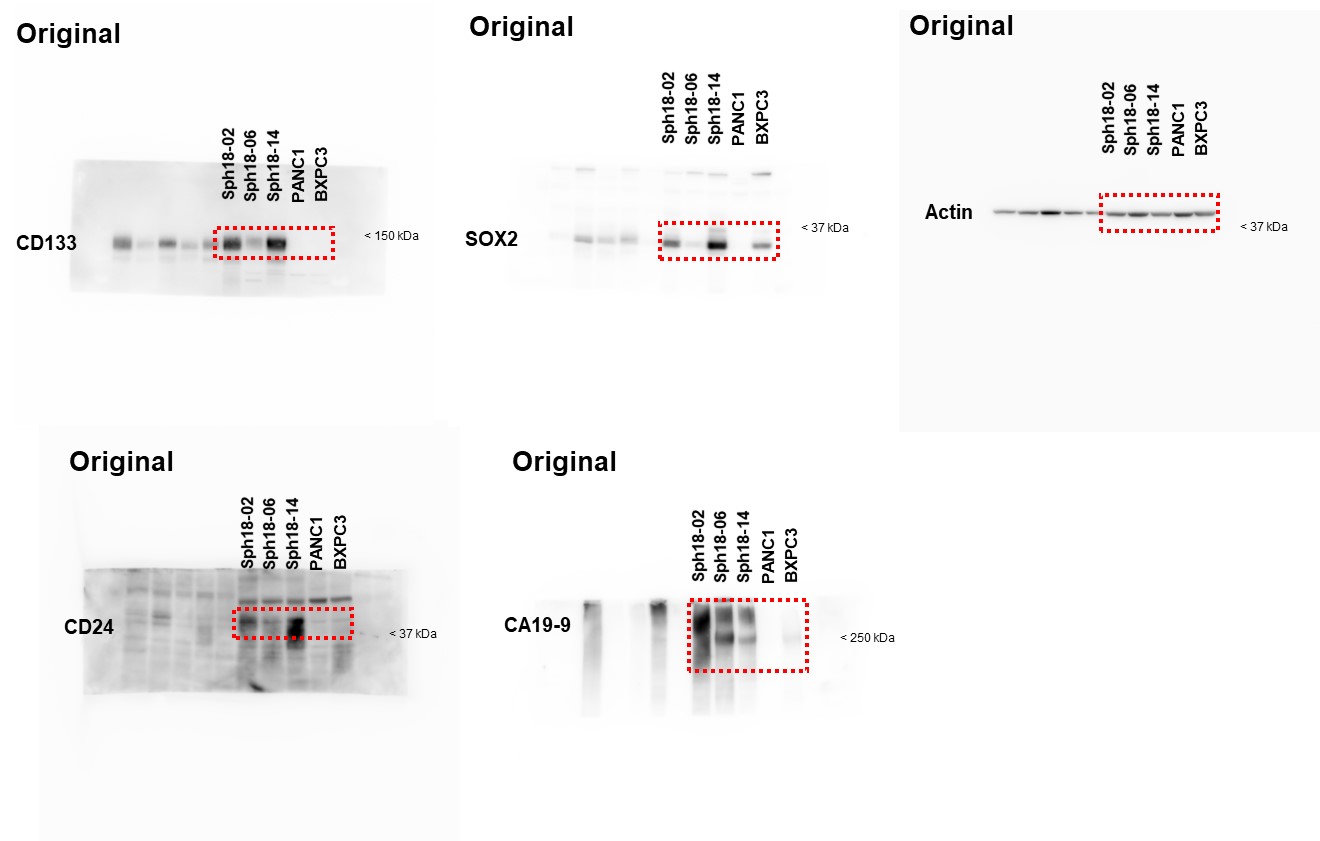
**
